# Factors associated with in-hospital death in patients with nosocomial infections: a registry-based study using community data in western Iran

**DOI:** 10.4178/epih.e2020037

**Published:** 2020-06-01

**Authors:** Salman Khazaei, Erfan Ayubi, Ensiyeh Jenabi, Saeid Bashirian, Masud Shojaeian, Leili Tapak

**Affiliations:** 1 Research Center for Health Sciences, Hamadan University of Medical Sciences, Hamadan, Iran; 2 Department of Community Medicine, School of Medicine, Zahedan University of Medical Sciences, Zahedan, Iran; 3 Autism Spectrum Disorders Research Center, Hamadan University of Medical Sciences, Hamadan, Iran; 4 Social Determinants of Health Research Center, Hamadan University of Medical Sciences, Hamadan, Iran; 5 Deputy of Health, Hamadan University of Medical Sciences, Hamadan, Iran; 6 Department of Biostatistics, School of Public Health, Hamadan University of Medical Sciences, Hamadan, Iran; 7 Noncommunicable Diseases Research Center, Hamadan University of Medical Sciences, Hamadan, Iran

**Keywords:** Nosocomial infections, Survival, Mortality, Iran

## Abstract

**OBJECTIVES:**

Determining the predictors of in-hospital death related to nosocomial infections is an essential part of efforts made in the overall health system to improve the delivery of health care to patients. Therefore, this study investigated the predictors of in-hospital death related to nosocomial infections.

**METHODS:**

This registry-based, longitudinal study analyzed data on 8,895 hospital-acquired infections (HAIs) in Hamadan Province, Iran from March 2017 to December 2019. The medical records of all patients who had been admitted to the hospitals were extracted from the Iranian Nosocomial Infections Surveillance Software. The effects of the type and site of infection, as well as age group, on in-hospital death were estimated using univariate and multivariable Cox regression models.

**RESULTS:**

In total, 4,232 (47.8%) patients with HAIs were males, and their mean age was 48.25±26.22 years. In both sexes, most nosocomial infections involved Gram-negative bacteria and the most common site of infection was the urinary tract. Older patients had a higher risk of in-hospital death (adjusted hazard ratio [aHR], 2.26; 95% confidence interval [CI], 1.38 to 3.69 for males; aHR, 2.44; 95% CI, 1.29 to 4.62 for females). In both sexes, compared with urinary tract infections, an increased risk of in-hospital death was found for ventilator-associated events (VAEs) (by 95% for males and 93% for females) and bloodstream infections (BSIs) (by 67% for males and 82% for females).

**CONCLUSIONS:**

We found that VAEs, BSIs, and fungal infections were independently and strongly associated with increased mortality.

## INTRODUCTION

Nosocomial infections (NIs) are among the leading causes of mortality and morbidity in hospitals, affecting hundreds of millions of patients around the world [1]. NIs increase hospital costs through the additional use of drugs—especially antibiotics—and by increasing the length of patients’ stay in the hospital [2].

The World Health Organization defines NIs as infections occurring in a patient in a hospital or other healthcare facility, in whom the infection was not present nor incubating at the time of admission, as well as infections that are acquired during the stay but appear after discharge [3].

Globally, a huge number of patients experience NIs, with incidence rates ranging from 3.5% to 12.0% in developed countries and from 5.7% to 19.1% in low-income and middle-income countries [4]. In a 1-day point prevalence study involving 1,265 intensive care units (ICUs) from 76 countries (the Extended Prevalence of Infection in Intensive Care study), 51% of patients were found to have NIs [5]. However, the variation reported in the literature can be attributed to the setting, the type of hospital, the patient population, and the precise definitions and surveillance techniques used in different societies [6]. In developing countries, the rates of nosocomial infections are over 8 times higher than in ICUs in the United States [7]. Evidence shows that the implementation of effective programs regarding NI surveillance can reduce the infection by approximately one-third [8].

It has been reported that bacteremia, lung disease, and multisite infection, as well as pneumonia in intensive care patients, are associated with an increased risk of mortality in patients with NIs [9,10]. The results of a multicenter study in France showed that 39%, 20%, and 14% of all NIs were attributed to lower respiratory tract, bloodstream, and surgical wound infections, respectively [11]. According another study in Norway, bloodstream infections (BSIs), lower respiratory tract infections, and multiple simultaneous infections were predictors of death in patients with NIs [12].

Carrying out studies to determine the predictors of mortality and to analyze serious untoward events during the provision of health services is an essential part of the efforts made by the overall health system to improve the delivery of health care to patients. Therefore, the aim of this study was to investigate the predictors of in-hospital NI-associated death.

## MATERIALS AND METHODS

This registry-based, longitudinal study was conducted using data on 8,895 hospital-acquired infections (HAIs) in Hamadan Province in western Iran from March 2017 to December 2019. Hamadan Province encompasses an area of 19,493 km^2^ and had a population of 1,758,268 people according to the national census held in 2016 by Statistical Center of Iran [13].

We used information on HAIs from the following hospitals: Alimoradian in Nahavand; Besat, Shahid-Beheshti, Sina, and Fatemieh in Hamadan; Vali-Asr in Tuyserkan; Ghaem in Asadabad; Imam Hossein and Mehr in Malayer; Imam Reza in Kabudarahang; Valiar in Razan; and Imam Reza in Kabudarahang, all of which recorded information on their infection rates in the Iranian Nosocomial Infections Surveillance Software (INISS).

Patients with clinical signs of an NI 48 hours after admission with no sign of bacterial colonization at the time of admission were enrolled in the study. The medical records of all patients who had been admitted to the hospital in the abovementioned time period were extracted from the INISS. We abstracted data from the software and recorded information including the demographic characteristics of patients (e.g., age and sex), year of occurrence, type of infection (Gram-negative bacterial, Gram-positive bacterial, viral, or fungal), site of infection (bone and joint; cardiovascular system; central nervous system; eye, ear, nose, or throat; gastrointestinal system; reproductive tract; and lower respiratory tract), and the ward of admission. Patients’ information was entered into the INISS daily, based on the findings of a physical examination and the patient’s signs and symptoms. The completeness and accuracy of the medical records was assessed by the infection control staff of the corresponding hospitals.

### Statistical analysis

The descriptive results are expressed as frequencies (%) or as mean±standard deviation. The survival of patients with NIs according to the type and site of infection was investigated using Kaplan-Meier survival analysis. The log-rank test was applied to evaluate the statistical significance of differences in survival curves. The effects of the type and site of infection, as well as age groups, on in-hospital death were estimated using univariate and multivariable Cox regression models and the results presented as hazard ratios (HRs) and 95% confidence intervals (CIs). The proportional hazards (PH) assumption of the variables included in the model was assessed using Schoenfeld residuals. A p-value < 0.05 was considered to indicate statistical significance. All statistical analyses were performed using Stata version 14 (StataCorp., College Station, TX, USA).

### Ethics statement

This study was approved by Ethics Committee of Hamadan University of Medical Sciences (IR.UMSHA.REC.1399.012).

## RESULTS

**Figure 1.**
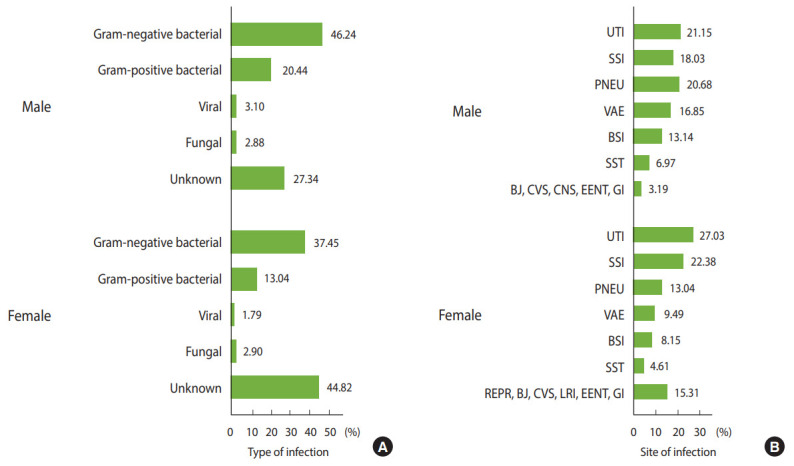
The distribution of nosocomial infections by (A) type and (B) site according to sex. UTI, urinary tract infection; SSI, surgical site infection; PNEU, pneumonia; VAE, ventilator-associated event; BSI, bloodstream infection; SST, skin and soft tissue; BJ, bone and joint; CVS, cardiovascular system; CNS, central nervous system; EENT, eye, ear, nose, throat, or mouth; GI, gastrointestinal; RPER, reproductive tract; LRI, lower respiratory infection.

In total, 8,895 patients with NIs were identified, of whom 4,232 (47.8%) were males (Table 1). Their mean age was 48.25±26.22 years (range, < 1 to 99). The mean length of hospital stay was 21.26±38.95 days (range, < 1 to 439) and the corresponding figures for time of admission to HAI and from HAI to discharge or death were 12.46±27.94 days (range, < 1 to 390) and 10.97±24.39 days (range, < 1 to 375), respectively. In both sexes, most NIs resulted from Gram-negative bacteria, followed by the Gram-positive bacteria and viral and fungal infections. By site, the most common infections were urinary tract infections (UTIs), followed by surgical site infections (SSIs), pneumonia (PNEU), ventilator-associated events (VAE), BSIs, skin and soft tissue infections, and infections of other organs (Figure 1). The distribution of NIs by type and site in both sexes according to age group are presented in Supplementary Materials 1 and 2.

**Figure 2.**
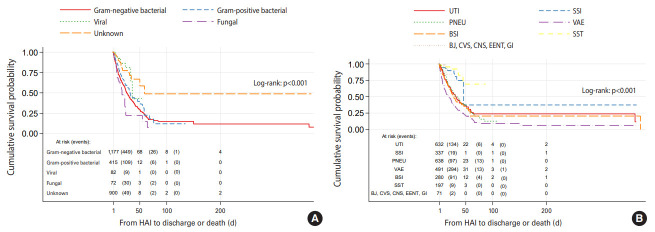
Kaplan-Meier curves of survival for hospital-acquired infections (HAI) in males according to (A) type and (B) site of infection. UTI, urinary tract infection; SSI, surgical site infection; PNEU, pneumonia; VAE, ventilator-associated event; BSI, bloodstream infection; SST, skin and soft tissue; BJ, bone and joint; CVS, cardiovascular system; CNS, central nervous system; EENT, eye, ear, nose, throat, or mouth; GI, gastrointestinal.

**Figure 3.**
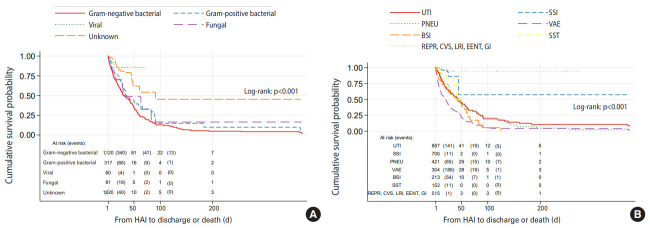
Kaplan-Meier curves of survival for hospital-acquired infections (HAI) in females according to (A) type and (B) site of infection. UTI, urinary tract infection; SSI, surgical site infection; PNEU, pneumonia; VAE, ventilator-associated event; BSI, bloodstream infection; SST, skin and soft tissue; CVS, cardiovascular system; CNS, central nervous system; EENT, eye, ear, nose, throat, or mouth; GI, gastrointestinal; RPER, reproductive tract; LRI, lower respiratory infection.

The effects of the type and site of HAIs on survival, as well as the results of sex-specific univariate Cox regression analyses, are shown in Table 2, Figures 2 and 3. Between March 2017 and December 2019, among 1,283 (20.5%) in-hospital deaths, 717 (55.8%) were in male patients and 566 (44.2%) were in female patients. Compared to Gram-negative bacterial infection, the crude HR of fungal infections for death was 1.40 (95% CI, 0.98 to 2.01; p=0.06) (Table 2). Applying Kaplan-Meier curves, survival was poorer in male cases with nosocomial fungal infections than in those with other types of infections (log-rank test, p<0.001) (Figure 2A), as the median survival for fungal infections was 17days (95% CI, 11 to 23), whereas the corresponding figure for Gram-negative bacterial infections was 26 days (95% CI, 23 to 30) (Figure 2A). Among male patients, the HR (95% CI) of VAEs for mortality compared to UTIs was 1.80 (95% CI, 1.47 to 2.19; p<0.001) (Table 2). Male with VAEs had shorter survival than those with other infections (log-rank test, p<0.001) with a median survival of 18 days (95% CI, 14 to 22) (Figure 2B).

Female patients with fungal and Gram-negative bacterial NIs had significantly worse survival than those with other types of infections, with a median survival of 31 days (95% CI, 27 to 83) and 32 days (95% CI, 27 to 40), respectively (log-rank test, p<0.001) (Figure 3A). Similarly to male patients, female patients with VAEs had significantly shorter survival than those with NIs at other sites (log-rank test, p<0.001) with a median survival of 20 days (95% CI, 14 to 24) (Figure 2B). Compared to UTIs, the crude HR for mortality associated with VAEs was 1.93 (95% CI, 1.56 to 2.39; p<0.001) (Table 2). The Schoenfeld residuals test did not suggest violation of the PH assumption for the variables included in the analysis.

The results of the multivariable Cox regression analysis are presented in Table 2. Older patients (> 65 years) had a higher risk of in-hospital death than younger patients (15-24 years), with aHRs of 2.26 (95% CI, 1.38 to 3.69) for males and 2.44 (95% CI, 1.29 to 4.62) for females. Among male patients with fungal NIs, an 87% increase was found in the risk of in-hospital death when compared to those with Gram-negative bacterial infections. Among both sexes, compared with UTIs, an increased risk of in-hospital death was found for VAEs (by 95% for males and 93% for females) and BSIs (by 67% for males and 82% for females). Although, SSIs were associated with a lower risk of in-hospital death—by 50% for males and 69% for females—these associations were not statistically significant.

## DISCUSSION

This study was conducted to identify the predictors of in-hospital death related to NIs. The main findings in this study were that in both sexes, bacteria were the most common cause of NIs. Moreover, by site, UTIs were most common, followed by SSIs and PNEU. Older patients had a higher risk of in-hospital death. Fungal NIs were associated with a higher risk of in-hospital death than Gram-negative bacterial infections in males. Among both sexes, compared with UTIs, VAEs, and BSIs were associated with a higher risk of in-hospital death, while SSIs were associated with a lower risk of in-hospital death.

Bacteria are responsible for roughly 90% of NIs, whereas other microorganisms make less of a contribution to the development of NIs [2]. This may be because some bacteria belong to the natural flora of the patient and cause an infection only when the immune system of the patient is weakened. We likewise found that bacteria were the leading cause of NIs in hospitalized patients in Hamadan Province. The results of the present study indicate that the most common NIs in inpatients of both sexes were UTIs. In 2007, an analysis of the INISS showed that UTIs were the most common NI in Iran, accounting for 32.2% of cases, but in that year, UTIs were most common in females, whereas PNEU was most common in males [14]. Differences in patterns of using invasive procedures for therapeutic and diagnostic purposes, implementing chemotherapy programs for cancer, and performing organ transplants in hospitals may cause differences in the distribution of NIs across sites and hospitals [15].

We found that in both sexes, most NIs resulted from Gramnegative bacteria. Gram-negative bacteria are bacteria that do not retain the crystal violet stain used in the Gram staining method of bacterial differentiation. Gram-negative bacilli cause the 4 most frequent types of HAI, including PNEU, SSIs, UTIs, and BSIs, which are caused by *Klebsiella, Acinetobacter, Pseudomonas aeruginosa*, and *Escherichia coli*, as well as many other less common bacteria [16]. Consistent with our findings, in the United States Gram-negative bacteria are responsible for more than 30% of HAIs, whereas in the ICUs of the United States, Gram-negative bacteria account for about 70% of these types of infections [17]. These bacteria are often resistant to multiple drugs and are increasingly resistant to most available antibiotics [18].

In accordance with our findings, Koch et al. [12] showed that old age was associated with an increased risk of death in patients with NIs. Defective host defenses and the prevalence of many chronic diseases such as cancer, diabetes, and atherosclerosis are partly responsible for the increased frequency of infections and death in older patients [19]. Differences in immunity status and other comorbidities can be associated with differences in the distribution of sites of infection according to age and, consequently, in the risk of death. Avci et al. [20] showed that the most common site of infection in elderly patients was the urinary tract, whereas in non-elderly patients it was the lower respiratory tract. The incidence of UTIs, respiratory tract infections, SSIs, skin and soft tissue infections, primary bacteremia, and prosthesis infections was significantly higher in elderly patients.

In accordance with our results, other studies have also shown that patients with BSIs had an increased risk of death [21,22]. We also found that the mortality risk for patients with SSIs was lower than others, a result that has also been observed in other studies [22,23].

The present study had some limitations. First, the sensitivity of the NI surveillance system in Iran is relatively low and does not cover all patients [14]. A second limitation of this study is the lack of access to denominator data to calculate the incidence of NIs by age group, sex, and microorganism type. Finally, it was not possible in this study to investigate illness severity as a possible predictor of death.

We found that VAEs, BSIs, and fungal infections were independently and strongly associated with increased mortality in hospitalized patients.

## Figures and Tables

**Table 1.** General characteristics for in-hospital deaths by age and sex according to the type and site of nosocomial infections

Characteristics	Male (n=4,232)		Female (n=4,627)	
	No (n=2,041)	Yes (n=717)	No (n=2,943)	Yes (n=566)
Age (yr)				
15-24	113 (5.5)	17 (2.4)	369 (12.5)	10 (1.8)
0-14	397 (19.4)	16 (2.2)	304 (10.3)	5 (0.9)
25-54	582 (28.5)	153 (21.3)	1,433 (48.7)	70 (12.4)
55-64	303 (14.8)	104 (14.5)	273 (9.3)	80 (14.1)
≥ 65	646 (31.6)	427 (59.5)	564 (19.2)	401 (70.8)
Type of infection				
Gram-negative bacterial	725 (35.5)	505 (70.4)	740 (25.1)	414 (73.1)
Gram-positive bacterial	308 (15.1)	119 (16.6)	250 (8.5)	80 (14.1)
Viral	79 (3.9)	9 (1.3)	59 (2.0)	4 (0.7)
Fungal	41 (2.0)	32 (4.5)	61 (2.1)	24 (4.2)
Unknown	888 (43.5)	52 (7.5)	1,833 (62.3)	44 (7.8)
Site of infection				
UTI	510 (25.0)	146 (20.4)	750 (25.5)	172 (30.4)
SSI	341 (16.7)	19 (2.6)	790 (26.8)	11 (1.9)
PNEU	534 (26.2)	113 (15.8)	339 (11.5)	91 (16.1)
VAE	186 (9.1)	320 (44.6)	96 (3.3)	213 (37.6)
BSI	190 (9.3)	108 (15.1)	156 (5.3)	67 (11.8)
SST	204 (10.0)	9 (1.3)	163 (5.5)	11 (1.9)
Other^1^	76 (3.7)	2 (0.3)	649 (22.0)	1 (0.2)

Values are presented as number (%). UTI, urinary tract infection; SSI, surgical site infection; PNEU, pneumonia; VAE, ventilator-associated events; BSI, bloodstream infection; SST, skin and soft tissue infection

1 Bone and joint, cardiovascular system, central nervous system, eye, ear, nose, throat, or mouth, and gastrointestinal infections for males; Reproductive tract, bone and joint, cardiovascular system, lower respiratory infections, and gastrointestinal infections for females.

**Table 2.** Hazard ratios for in-hospital death by age and sex according to the type and site of nosocomial infections

Variables		Male				Female			
		cHR (95% CI)	p-value	aHR (95% CI)	p-value	cHR (95% CI)	p-value	aHR (95% CI)	p-value
Age (yr)									
	15-24	1.00 (reference)		1.00 (reference)		1.00 (reference)		1.00 (reference)	
	0-14	0.51 (0.26, 1.02)	0.060	0.54 (0.27, 1.08)	0.080	0.45 (0.15, 1.32)	0.150	0.27 (0.09, 0.79)	0.020
	25-54	1.45 (0.88, 2.41)	0.140	1.40 (0.84, 2.32)	0.180	1.25 (0.64, 2.44)	0.500	1.02 (0.52, 1.99)	0.940
	55-64	1.81 (1.08, 3.04)	0.020	1.49 (0.89, 2.51)	0.130	3.86 (1.99, 7.46)	<0.001	2.06 (1.06, 4.01)	0.030
	≥ 65	2.79 (1.72, 4.53)	<0.001	2.26 (1.38, 3.69)	0.001	5.25 (2.79, 9.86)	<0.001	2.44 (1.29, 4.62)	0.006
Type of infection									
	Gram-negative bacterial	1.00 (reference)		1.00 (reference)		1.00 (reference)		1.00 (reference)	
	Gram-positive bacterial	0.78 (0.63, 0.95)	0.020	0.93 (0.75, 1.15)	0.520	0.72 (0.56, 0.91)	0.008	0.87 (0.67, 1.12)	0.290
	Viral	0.32 (0.16, 0.61)	0.001	0.87 (0.43, 1.76)	0.710	0.29 (0.11, 0.77)	0.010	0.81 (0.28, 2.27)	0.690
	Fungal	1.40 (0.98, 2.01)	0.060	1.87 (1.28, 2.74)	0.001	0.75 (0.48, 1.17)	0.210	1.11 (0.71, 1.75)	0.630
	Unknown	0.31 (0.23, 0.41)	<0.001	0.58 (0.42, 0.81)	0.001	0.17 (0.13, 0.24)	<0.001	0.59 (0.40, 0.86)	0.007
Site of infection									
	UTI	1.00 (reference)		1.00 (reference)		1.00 (reference)		1.00 (reference)	
	SSI	0.31 (0.19, 0.50)	<0.001	0.50 (0.30, 0.83)	0.008	0.15 (0.08, 0.28)	<0.001	0.31 (0.16, 0.60)	0.001
	PNEU	0.86 (0.67, 1.10)	0.230	1.14 (0.87, 1.49)	0.330	0.96 (0.74, 1.24)	0.750	1.03 (0.78, 1.36)	0.810
	VAE	1.80 (1.47, 2.19)	<0.001	1.94 (1.57, 2.40)	<0.001	2.15 (1.75, 2.64)	<0.001	1.93 (1.56, 2.39)	<0.001
	BSI	1.17 (0.90, 1.52)	0.220	1.67 (1.26, 2.22)	<0.001	1.40 (1.04, 1.87)	0.020	1.82 (1.34, 2.46)	<0.001
	SST	0.20 (0.10, 0.40)	<0.001	0.40 (0.20, 0.83)	0.010	0.46 (0.25, 0.85)	0.010	0.87 (0.45, 1.71)	0.700
	Other^1^	0.25 (0.06, 1.01)	0.050	0.48 (0.11, 1.99)	0.310	0.02 (0.00, 0.15)	<0.001	0.06 (0.00, 0.45)	0.006

cHR, crude hazard ratio; aHR, adjusted hazard ratio; UTI, urinary tract infection; SSI, surgical site infection; PNEU, pneumonia; VAE, ventilator-associated events; BSI, bloodstream infection; SST, skin and soft tissue infection

1 Bone and joint, cardiovascular system, central nervous system, eye, ear, nose, throat, or mouth, and gastrointestinal infections for males; Reproductive tract, bone and joint, cardiovascular system, lower respiratory infections, and gastrointestinal infections for females.
