# Supplementary material for: Factors associated with in-hospital death in patients with nosocomial infections: a registry-based study using community data in western Iran
Source: Epidemiol Health. 2020 Jun 1;42:e2020037. doi: 10.4178/epih.e2020037 (PMC7644946; doi:10.4178/epih.e2020037)
Supplement: Supplementary Material 2. [file epih-42-e2020037-suppl2.pdf]

Volume: 42, Article ID: e2020037

https://doi.org/10.4178/epih.e2020037

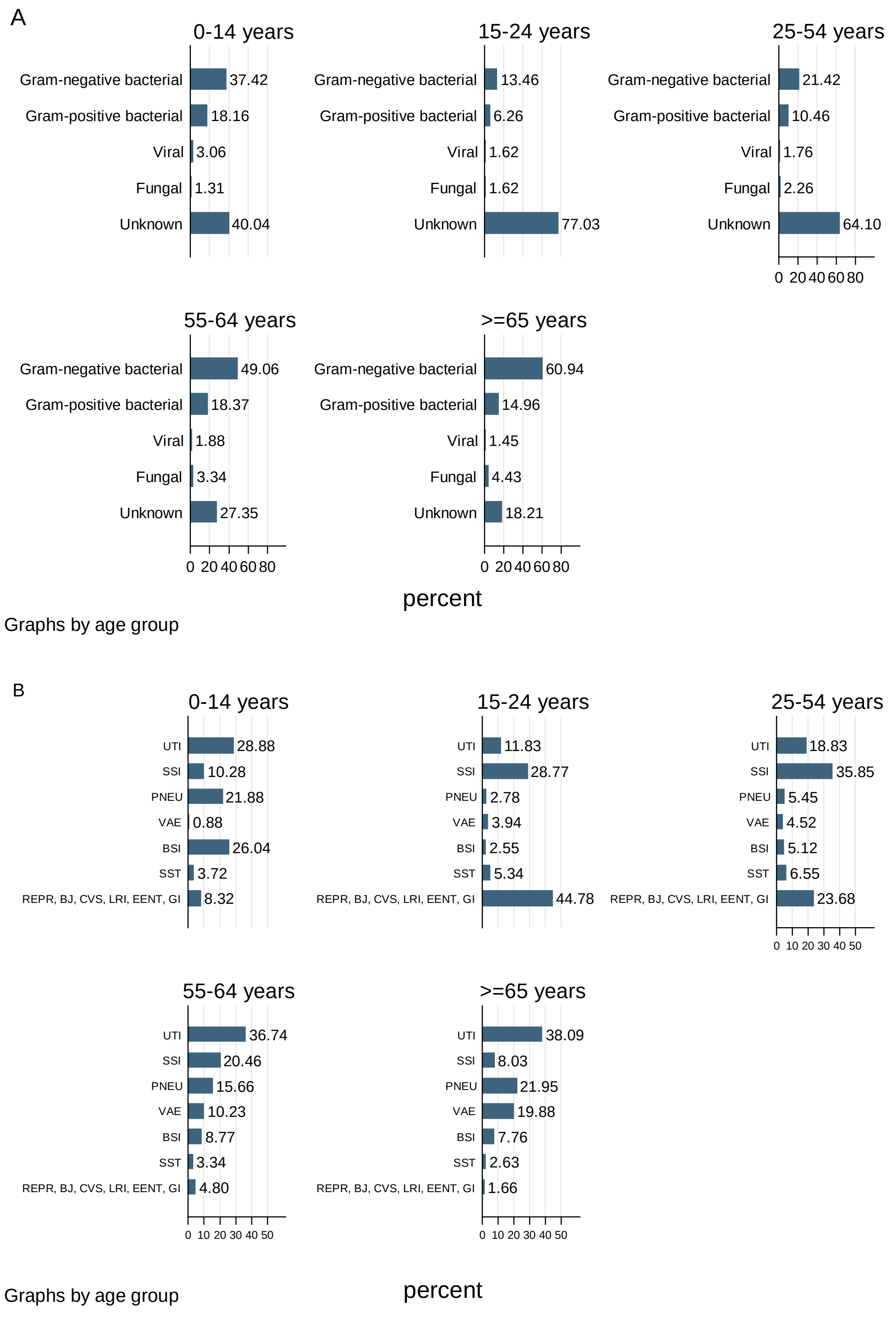

Supplementary Material 2. The percent of type (A) and site (B) of nosocomial infection in females. UTI: urinary tract infection, SSI: Surgical site infection, PNEU: pneumonia, VAE: ventilator-associated events, BSI: bloodstream infection, SST: Skin and Soft Tissue Infection BJ; Bone and Joint, RPER; Reproductive Tract, CVS; Cardiovascular System, LRI; Lower Respiratory, EENT; Eye, Ear, Nose, Throat, or Mouth, GI; Gastrointestinal infection.
